# Effects of Zearalenone on Production Performance, Egg Quality, Ovarian Function and Gut Microbiota of Laying Hens

**DOI:** 10.3390/toxins14100653

**Published:** 2022-09-21

**Authors:** Tao Yuan, Junyi Li, Yanan Wang, Meiling Li, Ao Yang, Chenxi Ren, Desheng Qi, Niya Zhang

**Affiliations:** Department of Animal Nutrition and Feed Science, College of Animal Science and Technology, Huazhong Agricultural University, Wuhan 430070, China

**Keywords:** laying hen, zearalenone, production performance, ovarian function, gut microbiota

## Abstract

Zearalenone (ZEN) is a ubiquitous contaminant in poultry feed, since ZEN and its metabolites can interfere with estrogen function and affect the reproductive ability of animals. The estrogen-like effect of ZEN on mammal is widely reported, while little information is available, regarding the effect of relatively low dose of ZEN on estrogen function and production performance of laying hens, and the relationship between them. This work was aimed to investigate the effects of ZEN on the production performance, egg quality, ovarian function and gut microbiota of laying hens. A total of 96 Hy-line brown laying hens aged 25-week were randomly divided into 3 groups including basal diet group (BD group), basal diet supplemented with 250 μg/kg (250 μg/kg ZEN group) and 750 μg/kg (750 μg/kg ZEN group) ZEN group. Here, 750 μg/kg ZEN resulted in a significant increase in the feed conversion ratio (FCR) (g feed/g egg) (*p* < 0.05), a decrease in the egg production (*p* > 0.05), albumen height and Haugh unit (*p* > 0.05), compared to the BD group. The serum Follicle-stimulating hormone (FSH) levels significantly decreased in ZEN supplemented groups (*p* < 0.05). Serum Luteinizing hormone (LH) and Progesterone (P) levels in the 750 μg/kg ZEN group were significantly lower than those in the BD group (*p* < 0.05). 16S rRNA sequencing indicated that ZEN reduced cecum microbial diversity (*p* < 0.05) and altered gut microbiota composition. In contrast to 250 μg/kg ZEN, 750 μg/kg ZEN had more dramatic effects on the gut microbiota function. Spearman’s correlation analysis revealed negative correlations between the dominant bacteria of the 750 μg/kg ZEN group and the production performance, egg quality and ovarian function of hens. Overall, ZEN was shown to exert a detrimental effect on production performance, egg quality and ovarian function of laying hens in this study. Moreover, alterations in the composition and function of the gut microbiota induced by ZEN may be involved in the adverse effects of ZEN on laying hens.

## 1. Introduction

Zearalenone (ZEN), also known as F-2 toxin, is a secondary metabolite produced by fungi of the genus *Fusarium* [1]. Its widespread occurrence in moldy corn, wheat, barley, oats and rice renders it as one of the dangerous mycotoxins endangering the feed safety in the global livestock and poultry industries [2,3]. According to the report, 85.2% of feed samples collected in the Chinese market were contaminated with ZEN, with average levels ranging from 35 to 1478 µg/kg [4]. The poultry industry in sub-Saharan Africa (SSA) also faces feed insecurity issues, such as Kenya, it was reported that the positive rates of ZEN detected in poultry feed ingredients and poultry feed were 83% and 100%, respectively, and the average levels of ZEN were 71.3 µg/kg and 103.4 µg/kg, partly [5]. Currently, ZEN has been shown to be hepatotoxic, immunotoxic, reproductive toxic and genotoxic, among which, reproductive toxicity is the most typical [6]. Increasing evidence suggests that ZEN and its metabolites exhibit various estrogenic toxicities due to their structural similarity to estradiol [7]. ZEN is toxic to gametogenesis and embryonic development in humans and animals [8], such as, the addition of 0.8, 4, 10, 40 ppm of ZEN to the diet of mice reduced the weight of the placenta and fetus [9]. ZEN can cause uterus enlargement, reproductive tract abnormalities, reproductive performance reduction and reproductive hormone dysfunction in female animals [10,11], as well as testicular atrophy and sperm concentration deterioration in males [12], which seriously restricts the development of animal husbandry. The adverse consequences of dietary ZEN consumption by pigs, cattle, sheep, and broilers have been extensively reported [13,14,15,16]. Poultry is more tolerant to ZEN, comparing to pigs, which may be related to the low absorption rate of ZEN, the rapid elimination of metabolites and the high proportion of β-ZOL produced by the liver [17]. But there are still limitations in reporting the effects on reproductive performance, and egg quality of laying hens. Therefore, it is necessary to understand the risk of ZEN on laying hens.

The gastrointestinal microbiota plays a critical role in maintaining animal health and reproductive performance [18]. Modulation of gut microbiota composition has been proven to alleviate several estrogen-regulated disease states [19]. Mycotoxin-induced disorders of the gut microbiota are related in the development of mycotoxicosis. Researches have shown that mycotoxins lead to the elimination of beneficial gut bacteria and enhancement of pathogenic bacteria, which negatively impairs the gut microbiota homeostasis [20,21]. Hence, it will be novel and fascinating to explore the relationship between changes of gut microbiota after exposure to ZEN and the decreased reproductive performance caused by ZEN.

Based on this, the aim of this study was to investigate the effects of different concentrations of ZEN on production performance, egg quality, ovarian function (ovarian index, ovarian pathological features and serum reproductive hormones) and gut microbiota of laying hens, and to further explore the potential mechanisms of ZEN caused decrease in reproductive performance and damage to ovary of laying hens and there from the perspective of gut microbiota. The findings of this study may open a new avenue for understanding the mechanisms responsible for the adverse effects of ZEN on laying hens, and provide new insights into the prevention and control of ZEN hazards on laying hens.

## 2. Results

### 2.1. Effects of ZEN on the Production Performance of Laying Hens

As shown in Table 1, no significant differences in egg production and feed intake were observed among the BD, BD + 250 μg/kg ZEN and BD + 750 μg/kg ZEN groups at the week 2 (*p* > 0.05). However, the basal diets supplemented with 750 μg/kg ZEN resulted in a significant increase in the FCR (g feed/g egg) compared to the BD group (*p* < 0.05). At the week 5, there were no significant change in egg production and feed intake for each group (*p* > 0.05). Compared to the BD group, the FCR was significantly higher in BD + 750 μg/kg ZEN group (*p* < 0.05). These findings indicated that high doses of ZEN may exacerbate the negative effects on production performance of laying hens.

### 2.2. Effects of ZEN on the Egg Quality of Laying Hens

The egg quality results for each group were presented in Table 2. Diets containing 250 μg/kg and 750 μg/kg ZEN had no significant effect on egg weight, egg index, yolk color, egg shell thickness and egg shell strength (*p* > 0.05). Nevertheless, the high ZEN-contaminated groups showed a decrease in albumen height and Haugh unit (*p* > 0.05) at the week 5, while yolk color and egg shell strength exhibited an increasing trend. The residues of ZEN in eggs were showed in Table 3. Compared with the control group, no residues of ZEN and metabolites were detected in BD + 250 μg/kg ZEN group and BD + 750 μg/kg ZEN group.

### 2.3. Effects of ZEN on the Ovarian Function of Laying Hens

**Figure 1:**
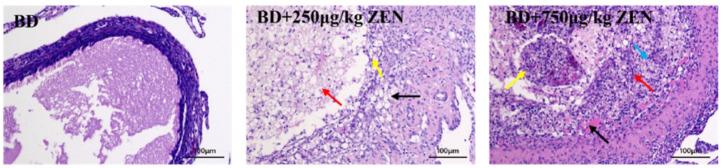
Effects of Zearalenone on histopathology in ovaries of laying hens. BD, base diet; BD + 250 μg/kg ZEN, base diet supplement with 250 μg/kg ZEN, black arrow: unclear structure of follicular granulosa cell layer and membrane cell layer, cell vacuolation degeneration, yellow arrow: cell necrosis degeneration, red arrow: few necrotic cells visible in follicular lumen; BD + 750 μg/kg ZEN, base diet supplement with 750 μg/kg ZEN, black arrow: protein mucus, blue arrow: unclear structure of follicular granulosa cell layer and membrane cell layer with markedly increased thickness, cellular vacuolation degeneration, red arrow: cellular necrosis degeneration, yellow arrow: cells shed in follicular lumen with some cellular necrosis. The bars represent 200 μm.

In this study, histological sections of ovarian tissue, ovarian index, oviductal index and serum reproductive hormone were administered to assess ovarian function. In contrast to the BD group, unclear structure of follicular granulosa cell layer and membrane cell layer was observed in the BD + 250 μg/kg ZEN group, along with the presence of a small number of necrotic cells in the follicular lumen. In addition, the ovarian of the BD + 750 μg/kg ZEN group showed an unclear structure of follicular granulosa cell layer and membrane cell layer with markedly increased thickness, and a large number of cells in the follicular lumen were shed and accompanied by partial cell necrosis (Figure 1). As shown in Table 4, compared to the BD group, the ZEN supplemented group showed a decreasing trend (*p* > 0.05) in body weight and ovarian index. ZEN had no significant effect on the Oviductal index (*p* > 0.05). The serum levels of E, FSH, LH and P are displayed in Table 5. The levels of E in the serum among the three groups revealed no significant differences (*p* > 0.05), whereas ZEN significantly reduced serum FSH levels compared to the BD group (*p* < 0.05). For serum LH, supplementation of 250 μg/kg ZEN in the diet had no significant influence (*p* > 0.05), however, serum LH levels in the BD + 750 μg/kg ZEN group were significantly lower than those in the BD group (*p* < 0.05). Meanwhile, serum P levels were significantly decreased in both the BD + 250 μg/kg ZEN group (*p* < 0.05) and the BD + 750 μg/kg ZEN group (*p* < 0.05) compared to the BD group.

### 2.4. Effects of ZEN on the Gut Microbiota of Laying Hens 

**Figure 2:**
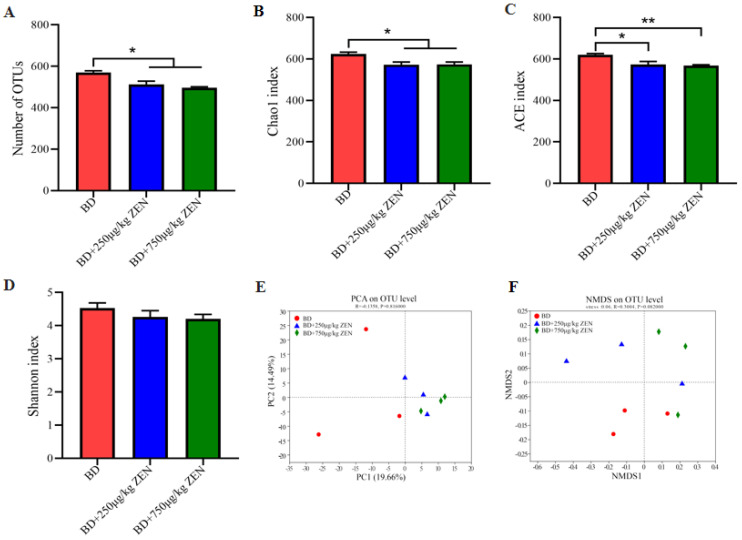
Effects of Zearalenone on 𝛼 and 𝛽 diversity of gut microbiota in laying hens. (**A**) Number of OTU, (**B**–**D**) α diversity of gut bacteria based on Chao1 index (**B**), ACE index (**C**) and Shannon index (**D***)*, (**E**) PCA plot of *β* diversity on OTU level, (**F**) NMDS on OUT level based on bray curtis. *, *p* < 0.05, **, *p* < 0.01. Note: *α* diversity is a measure of how diverse an individual’s microbiome is. 𝛽 diversity is an indicator to characterize similarity in microbial composition between individuals.

**Figure 3:**
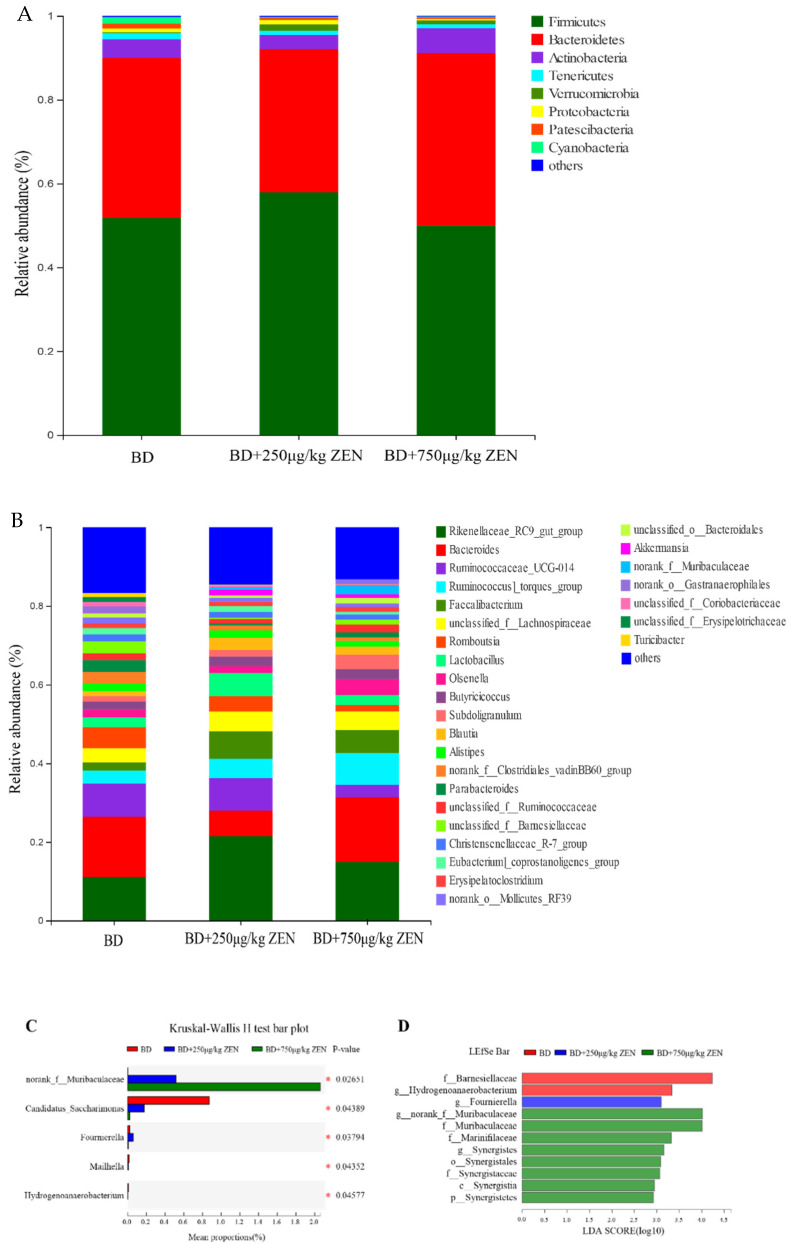
Effects of Zearalenone on the composition of gut microbiota in laying hens. (**A**) Relative abundance of phyla in gut microbiota, (**B**) Relative abundance of genera in gut microbiota, (**C**) Comparison of dominant genus among the three groups, (**D**) LEfSe bar based on phylum to genus level (LDA > 2). *, *p* < 0.05.

Figure 2 demonstrates the effect of different concentrations of ZEN on the α-diversity and β-diversity of cecum bacteria. As shown in Figure 2A, the dietary addition of 250 μg/kg ZEN and 750 μg/kg ZEN markedly reduced the number of OTUs of cecum bacteria compared to the BD group (*p* < 0.05). Cecum bacterial diversity was significantly lower (*p* < 0.05) in the ZEN-treated group as presented in the Chao1 index and ACE index (Figure 2B,C), while the Shannon index suggested a trend of reduced bacterial diversity (*p* > 0.05) (Figure 2D). The results of the principal component analysis (PCA) at the OTU level disclosed distinct differences in microbiota structure among the groups, and the non-metric multidimensional scaling (NMDS) based on bray curtis presented similar findings. Further, bacterial relative abundance of each group was appraised at the phylum and genus level, respectively (Figure 3).

As shown in Figure 3A, the highest relative abundance of Firmicutes was found in the intestinal flora of laying hens at the phylum level, which accounted for about 50–60%. The bacteria with the second highest relative abundance was Bacteroidetes, accounting for about 30–40%. The relative abundance of Actinobacteria, Tenericutes, Verrucomicrobia, Proteobacteria, Patescibacteria, and Cyanobacteria was at least greater than 1% in one of the groups. There were no significant differences in the abundance of bacteria among the groups at the phylum level (data not shown). Further analyzed at the genus level, 28 bacteria with relative abundance greater than 1% were observed (Figure 3B), where the relative abundance of norank_*f__Muribaculaceae* showed a dose-dependent significant increase in the ZEN supplementation group, while the opposite trend was observed in the relative abundance of Candidatus_Saccharimonas (Figure 3C). The LEfSe results at the phylum to genus levels shown in Figure 3D indicated that the dominant bacteria in the BD group were *f_Barnesiellaceae* and *g_Hydrogenoanaerobacterium*. And *g_Fournierella* was the predominant bacterium in the BD + 250 μg/kg ZEN group. The number of superior bacteria in the BD + 750 μg/kg ZEN group was significantly higher than the other two groups.

**Figure 4:**
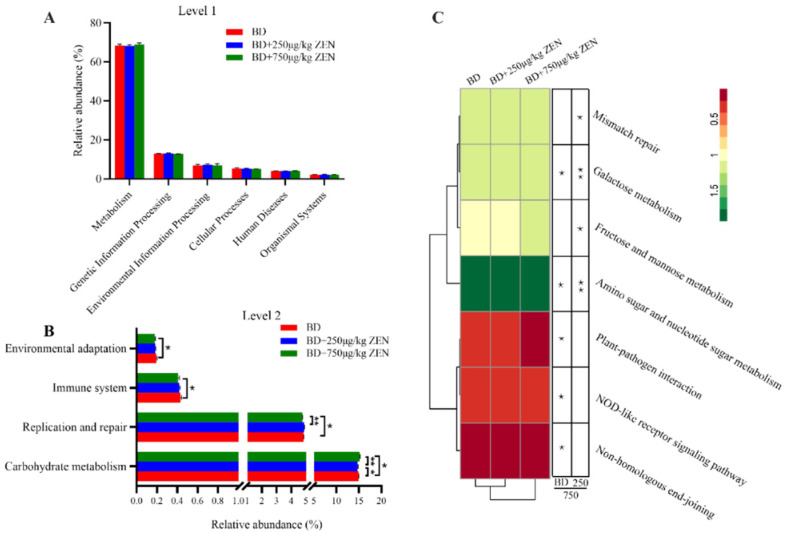
Effect of zearalenone on functional profiles of gut microbiota at KEGG level 1 (**A**), level 2 (**B**) and level 3 (**C**). *, *p* < 0.05, **, *p* < 0.01.

To investigate the influences of gut microbiota composition and abundance on the functional changes, the KEGG database was used for functional prediction. As shown in Figure 4A, ZEN had no significant effect on the 6 pathways at KEGG level 1. Interestingly, four pathways including carbohydrate metabolism, replication and repair, immune system and environmental adaptation were significantly different at KEGG level2 (Figure 4B). Additionally, 7 pathways were remarkably different at level 3 of these four pathways. The heatmap results showed that the BD and BD + 250 μg/kg ZEN groups were more functionally similar, indicating that higher concentrations of ZEN had a greater effect on the function of the gut microbiota (Figure 4C).

### 2.5. Correlation between Gut Microbiota and Reproductive Performance and Ovarian Function Faliure of Laying Hens

**Figure 5:**
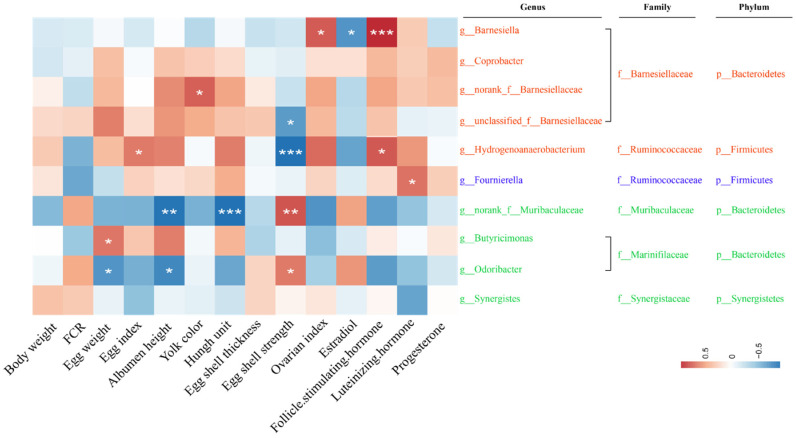
Correlation of gut microbiota with Zearalenone-induced impairment of production performance, egg quality (week 5) and ovarian function in laying hens. * *p* < 0.05, ** *p* < 0.01 and *** *p* < 0.001 indicate significant correlation.

Next, Spearman’s correlation analysis was applied to further examine the associations between the dominant bacteria and the ZEN-induced production performance reduction as well as ovarian function dysfunction in each group (Figure 5). The *g_Barnesiella* of the BD group showed a significant positive correlation with ovarian index and LSH level, while it showed a negative trend with production performance and egg quality indicators. As a whole, the other four dominant bacteria in the BD group at the genus level showed positive correlations with production performance, egg quality and ovarian function, except for *f_Barnesiellaceae* and *g_Hydrogenoanaerobacterium*, which showed remarkable negative correlations with egg shell strength. For the BD + 250 μg/kg ZEN group, the dominant bacteria *g_Fournierell* showed a meaningful positive correlation with LH, and none of the correlations with other indicators were significant. Intriguingly, the dominant bacteria in the BD + 750 μg/kg ZEN groups presented negative correlations with each indicator at large, with *g_Odoribacte* and *f_Muribaculaceae* showing major negative relationships with egg weight, albumen height and Haugh unit.

## 3. Discussion

The global problem of mycotoxin contamination in feed is very prominent, among which, ZEN is one of the common mycotoxins in feed [2]. ZEN is well known to induce non-negligible estrogenic effects in humans and animals owing to its estrogen-like toxicity, which contributes considerably in the harm of production performance and reproductive disorders in animals, especially in female animals [22,23]. The risk of ZEN on sows has been broadly studied and some reports related to the broiler chickens have been reported, while few works have been conducted on laying hens.

In our present study, ZEN had no significant undesirable influences on egg production and feed intake, which was in agreement with the results of Sypecka, et al. [24] who reported that the addition of 275 μg/kg of ZEN to the diet of 19-week-old Bovan Goldline laying hens for 3 weeks did not make a significant difference in feed intake and egg production. Unfortunately, the findings were in contrast to those of Dänicke, et al. [25], as they showed that 1508 μg/kg of ZEN fed continuously to 22-week-old Lohmann Brown laying hybrids for 16 weeks reduced feed intake by approximately 5%, as well as egg production and FCR. Similar results for egg production and feed intake were also obtained by Chowdhury and Smith [26]. The discrepancy between these results and previous reports may be intimately related to the feeding dose and feeding duration of ZEN. Nevertheless, the dietary addition of high doses of ZEN (750 μg/kg ZEN) significantly increased FCR in this study (*p* < 0.05). It was noted that the addition of 600 μg/kg of ZEN to the diet of 45-week-old laying hens for 12 weeks reduced the FCR compared to the control group [26]. In addition, this was also supported by the report of Dänicke, et al. [25]. Overall, these highlighted inconsistencies are probably attributed to dose- and time-dependent effects of ZEN on production performance of animals, which could also be derived by the variation in the absorption, transport and metabolism of ZEN among hens with different ages and breeds.

The external and internal quality of eggs are the principal components in evaluating the quality of eggs. The yolk color, albumen height and Haugh unit are regarded as parameters for assessing the internal quality of eggs, while the external quality of eggs consists of egg weight, egg index and egg shell quality including egg shell thickness and egg shell strength [27,28]. Mycotoxins are one of the numerous essential factors in affecting the egg quality [29,30]. In poultry industry, the problem of potential acute or chronic damage of ZEN on egg quality is easily overlooked, which is a direct response to the lack of studies on the effects of ZEN on egg quality. Jia, et al. [31] reported that the supplementation of 260.2 μg/kg ZEN in the diet of 18-week-old Hy-line brown hens for 5 weeks showed no significant differences in egg quality such as yolk color, albumen height, Haugh unit as well as egg shell strength, egg shell thickness and egg index compared to the control group. The findings of the present study with 250 μg/kg ZEN in the diet matched those of Jia, et al. [31]. Interestingly, high concentrations of ZEN (750 μg/kg) reduced albumen height and Haugh unit after 5 weeks of continuous feeding in our study. This result suggested that the impacts of ZEN on egg quality may be potentially related to the breed of hens, feeding strategy and feeding period. The residual amounts of ZEN and its metabolites in egg yolk were examined. No residues of ZEN and metabolites were found in eggs at a given zearalenone level and detection limit. This is consistent with Dänicke, et al’s result [32]. The reasons below the detection limit may be related to factors such as the ZEN metabolic site in the liver and the low content of added ZEN [25]. Given the limited reports on ZEN and egg quality, related studies should be carried out widely in the future.

ZEN and its metabolites exhibit estrogenic activity and compete for binding to the estradiol receptor contributing to weight and morphological changes in the reproductive organs and disorders of the reproductive system in animals [22,33,34]. Until now, most studies have focused on the effects of ZEN on reproductive performance in mammals, with few studies reporting its actions on ovarian function in laying hens. Ovarian function determines reproductive performance and egg quality [35], therefore, ovarian function of laying hens under ZEN treatment was further investigated. In this study, with the increase of ZEN concentration in the laying hen’s diet, the ovarian index decreased significantly and changes in ovarian structure were observed, mainly manifested by an ambiguous structure of follicular granulosa cell layer and membrane cell layer with markedly increased thickness, a visible shedding of cells in the follicular lumen and some cell necrosis. Similarly, the administration of 2.0 and 3.2 mg/kg of ZEN in the diet significantly reduced the number of primordial follicles and primary growing follicles in the ovarian cortex of weaned piglets. In particular, 3.2 mg/kg of ZEN further induced follicular atresia [36]. Zhao, et al. [37] found that 238 μg/kg of ZEN resulted in enlarged vulva size in pre-pubertal female gilts during a 24-d trial. In rats, 20 mg/kg of ZEN induced ovarian luteal cell vacuolization and significantly diminished the number of follicles [38]. Dong, et al. [39] administrated 2.4 mg/kg BW of ZEN intravenously to goats and observed a mild lymphocytic infiltration in the uterus 48 h later. The histopathological study in this experiment was consistent with the results of reproductive organ damage reported above, which all supported the risk of ZEN causing reproductive dysfunction. Furthermore, it was reasonable to assume that different doses of ZEN could elicit different degrees of reproductive organ damage in various species.

Owing to the fact that morphological and functional impairment of reproductive organs always accompanies with changes in reproductive hormone levels [40], we further examined the critical indicators E, FSH, LH and P to assess reproductive hormone levels [41,42]. In this study, ZEN had no significant effect on serum E level, but reduced serum FSH, LH and P levels in a dose-dependent manner. Serum E level was inconsistent with the results reported by Wang, et al. [43] who reported that ZEN significantly increased E level in sows, In contrast, it was reported that ZEN decreased the level of E in Kunming mice [44]. As shown in previous work, ZEN significantly reduced the levels of FSH, LH and P in prepubertal gilts [45], and similar results in rats were confirmed by Collins, et al. [46]. Unfortunately, studies on the effect of ZEN on reproductive hormone levels in laying hens were not covered. Taken together, the presence of 250 μg/kg or more of ZEN in the diet of laying hens will cause ovarian damage, which can be attributed to ZEN-induced endocrine and metabolic disorders. Moreover, ovarian atrophy caused by ZEN may be the main factor leading to poor egg quality.

Numerous studies have shown that gut microbiota contributes to a variety of metabolisms in the body and play a key role in maintaining intestinal homeostasis and host health [47,48]. We nextly discussed the effect of dietary exposure to ZEN on gut microbiota and its association with ZEN-induced depression of production performance, egg quality and ovarian function in laying hens. In the current study, we found that the relative abundance of *g__norank_f__Muribaculaceae* and *g_Odoribacter* was negatively correlated with egg quality and reproductive hormone levels following ZEN treatment, which suggested that these two bacteria may be involved in the metabolism of ZEN in the gut of laying hens and thus in the impacts of ZEN on laying hens. Obtained results also showed that high concentrations of ZEN altered the gut microbiota functions such as immune system and replication and repair. The microbiota functions of 250 μg/kg ZEN group were more similar to those of the BD group. This finding appears to support the idea that higher concentrations of ZEN have stronger detrimental effects on various indicators in laying hens. Piotrowska, et al. [49] first investigated the influence of ZEA on the gut microbiota in 2014 and showed that ZEN adversely affects the gut microbiota stability in pigs. Changes in metabolites elicited by alterations in the composition and structure of the gut microbiota further modulate the secretion of host reproductive hormone [50]. Accordingly, ZEN-induced shifts in gut microbiota probably modulate serum reproductive hormone levels in laying hens, thereby affecting egg quality and ovarian function. Overall, the studies on the role of gut microbiota in mycotoxin-induced health risks in animals are still scarce. Further researches using germ-free animals to demonstrate the association of mycotoxins with gut microbiota are warranted in the future.

## 4. Conclusions

In conclusion, our data revealed that 250 μg/kg ZEN had no significant effects on feed intake, egg production and egg quality, while 750 μg/kg ZEN significantly increased the FCR. In addition, the supplementation with 750 μg/kg ZEN decreased albumen height and Haugh unit. Both 250 μg/kg ZEN and 750 μg/kg ZEN resulted in ovarian index reduction, ovarian tissue damage and reproductive hormone dysregulation. In addition, the alteration of gut microbiota community structure by ZEN including the increase in relative abundance of *g__norank_f__Muribaculaceae* and *g_Odoribacter* may influence reproductive hormone levels. In this study, the association between the structural changes of gut microbiota and disorders of reproductive hormone secretion provided a new insight to elucidate the mechanism of ZEN-induced egg quality reduction and ovarian function impairment.

## 5. Materials and Methods

### 5.1. Animals, Experimental Design and Sample Collection

The Institutional Animal Care and Use Committee at Huazhong Agricultural University (Wuhan, China) approved the current animal experiments study. The Hy-line brown laying hens aged 23 weeks had stable egg production. At 25 weeks of age, the peak egg production period is reached, so 25-week-old laying hens were selected [51]. The reference doses for ZEN were set based on the results of Rong, et al. [9], Jia, et al. [31] and Chowdhury, et al. [52], combined with ZEN levels reported in feed [53,54,55]. A total of 96 Hy-line brown laying hens aged 25 weeks with similar body weight were randomly divided into 3 groups with 8 replicates each, and 4 hens in each replicate. Laying hens in the control group were provided with a corn soybean meal-based diets (BD group), in which the basal diet contained 60 μg/kg zearalenone, 8 μg/kg aflatoxin, and 800 μg/kg DON. The experimental groups were received basal diets supplemented with 250 μg/kg (BD + 250 μg/kg ZEN group) and 750 μg/kg (BD + 750 μg/kg ZEN group) ZEN (purity > 98%, Shanghai Yuanye Bio-Technology Co., Ltd, Shanghai, China), respectively. The composition and nutritional levels of the basal diet were shown in Table 6. Laying hens were housed in three-stage stepped-stainless steel cages with four hens per cage. After one week of acclimatization, the laying hens were provided with water and feed ad libitum and maintained in a controlled environment with a 16 h light/8 h dark cycle (temperature between 22 °C and 28 °C) during the five-week trial. Eggs were collected daily at 15:30 until the end of the trial. One laying hens from each replicate was randomly selected for blood collection and slaughter after 12 h of fasting (water only) at the end of the 5-week trial. Serum was collected after centrifugation at 3000 r/min for 10 min and stored at −20 °C for the determination of serum reproductive hormone levels. Ovarian tissue was fixed in 4% formaldehyde for the preparation of pathology sections. Other samples were collected and quickly frozen in liquid nitrogen followed by storage at −80 °C until further analysis.

### 5.2. Production Performance and Organ Index Analysis

The feed intake, number of hens and number of eggs were recorded daily. The average feed intake, feed conversion ratio (FCR, g feed/g egg) as well as egg production were calculated weekly. Live weight, ovarian and oviduct weights of slaughtered laying hens were noted for ovarian index and oviductal index analysis. Ovarian index = ovarian weigh (g)/live weight of laying hens (kg)*100%. Oviductal index = oviductal weigh (g)/live weight of laying hens (kg)*100%.

### 5.3. Egg Quality Analysis

Egg Quality Analysis was conducted as described in the previous study [56]. Eggs were collected in the 2nd and 5th week for analysis of egg quality including egg weight, yolk color, Haugh unit, albumen height, egg shell strength, egg shell thickness and egg index. Egg weight, yolk color, Haugh unit and albumen height were determined with a multifunctional egg tester (EMT-5200, Robotmation, Japan). Egg shell strength was analyzed with an egg shell strength tester (EFG-0503, Robotmation, Japan). The shell thickness of the egg was measured at the sharp end, equator and blunt end (excluding the inner shell membrane) using a spiral micrometer and the average of the three points was calculated to obtain the egg shell thickness. The egg index was evaluated by measuring the transverse diameter and the longitudinal diameter of the egg with vernier calipers. Egg index = longitudinal diameter/transverse diameter. ZEN, α-Zearalenol (α-ZOL) and β-Zearalenol (β-ZOL) residue in egg yolks was detected by immunoaffinity column cleanup-high performance liquid chromatography, according to You et al. [57]. The 5.00 g of egg yolk was weighed into a 50 mL centrifuge tube, adding to 10.0 mL of sodium acetate buffer and 25 μL of β-glucuronide/sulfate complex enzyme. After the samples were mixed, they were enzymatically hydrolyzed in a shaker at 37 °C for 12 h (100 r/min) and then cooled to room temperature. The samples were extracted with methyl tert-butyl ether, and subsequently reextracted with a sodium hydroxide solution. After the pH value was adjusted to 7, the extract was cleaned up on an immunoaffinity column. The Immunoaffinity column was IAC-SEP, and its column capacity was 1000 ng, volume 1mL, produced by Clover Technology Group Inc, Beijing, China. The chromatographic separation was performed on an Agilent XDB-C18 column (150 mm × 4.6 mm, 3.5 μm) using methanol-acetonitrile-water (50:15:35, *v*/*v*/*v*) as the mobile phase at a flow rate of 1.0 mL/min and ultraviolet (UV) detection at 270 nm.

### 5.4. Serum Reproductive Hormones Level Analysis

The reproductive hormones determined in serum include: Estradiol (E), Follicle-stimulating hormone (FSH), Luteinizing hormone (LH) and Progesterone (P). Serum reproductive hormones were performed as described by the manufacturer’s (Shanghai Jining Industrial Co., Ltd, Shanghai, China) instructions and measured using a Multiskan Sky microplate reader (Thermo Fisher Scientific, Waltham, MA, USA).

### 5.5. Morphology Analysis

For H&E staining of ovarian tissue, the ovarian tissue was dehydrated with graded alcohol (75%, 85%, 90%, 95% and 100%) and xylene (50% ethanol + 50% xylene, 100% xylene I and 100% xylene II) in sequence after 24 h of fixation, then embedded in paraffin, sectioned and stained with H&E staining. The prepared sections were observed under a Leica DM3000 microscope (LEICA, Wetzlar, Germany) for morphological and pathological features of the ovaries.

### 5.6. S rRNA Sequencing and Bioinformatics Analyses

The collected fresh cecum contents were instantly snap frozen in liquid nitrogen and stored at −80 °C [58] until used for enteric bacteria DNA extraction with the QIAamp DNA stool Mini Kit (Qiagen, Hilden, Germany). The V3-V4 region (338F: ACTCCTACGGGAGGCAGCA, 806R: GGACTACHVGGGTWTCTAAT) of the 16S rDNA was amplified by PCR, and the amplified products were examined by 2% gel electrophoresis. Afterwards, the assayed PCR products were recovered according to the axyprep DNA Gel Extraction Kit protocol (Axygen, Silicon Valley, CA, USA). A QuantiFluor™-ST microfluorometer (Promega, Madison, WI, USA) was used to detect and quantify PCR products, followed by construction of Miseq libraries for pyrosequencing on an Illumina MiSeq platform (Illumina, San Diego, CA, USA). Based on similarity ≥ 97%, clean sequences were distributed to the same operational taxonomic unit (OTU). Bioinformatics Analyses were performed on the free Majorbio (Shanghai, China) online cloud platform (https://cloud.majorbio.com/), whose access time was on 25 October 2021. β-diversity analyses were exhibited by PCoA plots and NMDS plots at the OTU level based on weighted unifrac indices and bray Curtis, respectively. The Kruskal-Wallis rank sum test was used to check the significant differences in relative abundance at the phylum and genus levels. The potential relationship between zearalenone-induced cecum microbiota and production performance of laying hens was initially revealed by Spearman’s correlation analysis after using Kolmogorov-Smirnova and Shapiro Wilk tests for normality distribution of data.

### 5.7. Statistical Analysis

Data were initially processed by Excel (Microsoft Office 2010, Seattle, WA, USA) and then analyzed by one-way ANOVA using SPSS 19.0 (SPSS, Inc., Chicago, IL, USA) software. Duncan’s multiple analysis of variance was used to compare the means among groups on the basis of significant ANOVA. *p* < 0.05 was considered statistically significant. Data were expressed as “mean ± standard deviation”.

## Figures and Tables

**Table 1:** Effects of Zearalenone on Production Performance of Laying Hens.

Week	Items	Supplemental Levels of ZEN in Diets, μg/kg		
		BD	BD + 250 μg/kg ZEN	BD + 750 μg/kg ZEN
Week 2	Egg production (%)	98.6 ± 2.0	98.6 ± 1.8	96.4 ± 3.2
	Feed intake (%)	109.9 ± 1.3	110.2 ± 1.7	109.8 ± 1.3
	FCR (g feed/g egg)	1.86 ± 0.09 ^b^	1.88 ± 0.04 ^b^	1.99 ± 0.15 ^a^
Week 5	Egg production (%)	98.3 ± 1.6	98.5 ± 1.0	96.6 ± 2.0
	Feed intake (%)	109.2 ± 1.1	109.4 ± 2.1	109.7 ± 0.8
	FCR (g feed/g egg)	1.83 ± 0.07 ^b^	1.86 ± 0.04 ^ab^	1.91 ± 0.08 ^a^

Values are expressed as means ± SD (*n* = 8), ^a,b^ means values with unlike letters were significantly different (*p* < 0.05).

**Table 2:** Effects of Zearalenone on Egg Quality of Laying Hens.

Week	Items	Supplemental Levels of ZEN in Diets, μg/kg		
		BD	BD + 250 μg/kg ZEN	BD + 750 μg/kg ZEN
Week 2	Egg weight (g)	62.0 ± 1.5	60.6 ± 1.7	60.6 ± 1.4
	Egg index	1.26 ± 0.04	1.27 ± 0.03	1.28 ± 0.02
	Albumen height (mm)	7.15 ± 1.62	7.40 ± 1.07	6.56 ± 0.60
	Yolk color	7.58 ± 1.73	8.75 ± 1.22	8.45 ± 2.14
	Haugh unit	83.92 ± 9.3	86.01 ± 6.3	80.67 ± 4.3
	Egg shell thickness (mm)	0.31 ± 0.02	0.32 ± 0.01	0.32 ± 0.02
	Egg shell strength (N)	44.7 ± 5.5	44.3 ± 7.3	47.6 ± 2.8
Week 5	Egg weight (g)	61.0 ± 2.0	60.4 ± 2.0	60.6 ± 2.9
	Egg index	1.26 ± 0.03	1.27 ± 0.03	1.27 ± 0.01
	Albumen height (mm)	6.66 ± 0.66	6.30 ± 1.10	5.85 ± 0.73
	Yolk color	8.11 ± 0.92	8.37 ± 0.62	9.26 ± 1.75
	Haugh unit	80.75 ± 4.0	76.84 ± 8.1	74.85 ± 5.3
	Egg shell thickness (mm)	0.33 ± 0.01	0.33 ± 0.01	0.32 ± 0.01
	Egg shell strength (N)	42.9 ± 5.9	42.0 ± 6.6	44.5 ± 4.5

Values are expressed as means ± SD (*n* = 8).

**Table 3:** Effect of residues of Zearalenone, α-ZOL, β-ZOL in yolks.

Items	Treatment Level		
	BD	BD + 250 μg/kg ZEN	BD + 750 μg/kg ZEN
ZEN	<LOD	<LOD	<LOD
α-ZOL	<LOD	<LOD	<LOD
β-ZOL	<LOD	<LOD	<LOD

Limit of detection (LOD) of ZEN, α-ZOL and β-ZOL are all 1 μg/kg.

**Table 4:** Effects of Zearalenone on Ovarian Index and Oviductal Index of Laying Hens.

Items	Supplemental Levels of ZEN in Diets, μg/kg		
	BD	BD + 250μg/kg ZEN	BD + 750 μg/kg ZEN
Body weight (kg)	1.96 ± 0.12	1.92 ± 0.15	1.89 ± 0.11
Ovarian index	0.26 ± 0.07	0.22 ± 0.03	0.21 ± 0.09
Oviductal index	3.29 ± 0.25	2.96 ± 0.19	3.10 ± 0.42

Values are expressed as means ± SD (*n* = 8).

**Table 5:** Effects of Zearalenone on Reproductive Hormones of Laying Hens.

Items	Supplemental Levels of ZEN in Diets, μg/kg		
	BD	BD + 250 μg/kg ZEN	BD + 750 μg/kg ZEN
Estradiol (pg/mL)	449.28 ± 44.90	440.72 ± 19.80	451.08 ± 43.26
Follicle-stimulating hormone (IU/L)	7.62 ± 0.31 ^a^	6.63 ± 0.33 ^b^	6.46 ± 0.25 ^b^
Luteinizing hormone (ng/mL)	151.85 ± 12.41 ^a^	145.84 ± 12.44 ^a^	129.35 ± 6.72 ^b^
Progesterone (pg/mL)	485.78 ± 113.40 ^a^	359.67 ± 64.04 ^b^	286.56 ± 57.40 ^b^

Values are expressed as means ± SD (*n* = 6), ^a,b^ means values with unlike letters were significantly different (*p* < 0.05).

**Table 6:** Basic diet components and nutrients.

Ingredients	%	Nutrition Component	Content (%)
Corn	51.90	Moisture	9.68
Soybean meal	16.00	Crude protein	16.05
Distillers Dried Grains with soluble (crude protein: 27%)	11.72	ME (MJ/kg)	11.28
Extruded soybean	4.00	Crude Ash	14.24
Corn germ meal	2.50	Crude fat	5.52
Soybean oil	2.43	Crude fiber	4.28
Shell powder	3.00	Calcium	3.76
Limestone	6.55	Phosphorus	0.52
Monocalcium phosphate	0.72	Lysine	0.85
Phytase	0.02	Methionine	0.48
Premix ^a^	1.10	Arginine	0.91
Total	99.4		

Premixe provided per kg of diet: Vitamin K3 (MSB), 6.8 mg; Vitamin B1, 3.26 mg; Vitamin A, 6600 IU; Vitamin D3, 3240 IU; Vitamin E, 60 mg; Vitamin B2, 11.25 mg; calcium pantothenate, 13.05 mg; niacinamide, 42.96 mg; Vitamin B6, 5.46 mg; biotina, 0.9 mg; folic acid, 1.22 mg; Iron, 99 mg; Manganese, 120 mg; Zinc, 100 mg. The ME in the basal diet was calculated and the content of other nutrients was measured. ^a^ Generally, in animal experiments, the metabolizable energy values of diets are calculated values, except in experiments about evaluation of feeds nutritional value.

## Data Availability

Sequence data supporting the results of this study are available in the NCBI Sequence Read Archive (SRA Biological Project Numbers: PRJNA782769).
